# Compliance and Adherence to Pelvic Floor Exercise Therapy in People with Pelvic Floor Disorders: A Systematic Review and Meta-Analysis

**DOI:** 10.3390/life15040613

**Published:** 2025-04-06

**Authors:** Inmaculada Villa-Del-Pino, José-Jesús Jiménez-Rejano, Manuel Rebollo-Salas, Álvaro-José Rodríguez-Domínguez, Carmen-María Suárez-Serrano

**Affiliations:** 1 Department of Physiotherapy, University of Seville, 41009 Seville, Spain; ivilla@centrosanisidoro.es (I.V.-D.-P.); jjjimenez@us.es (J.-J.J.-R.); csuarez@us.es (C.-M.S.-S.); 2 Department of Health and Sports, Pablo de Olavide University, 41013 Seville, Spain; arodriguezdominguez@centrosanisidoro.es

**Keywords:** exercise therapy, pelvic floor disorders, quality of life, urinary incontinence, sexual dysfunction, physiological, treatment adherence and compliance, health, musculoskeletal diseases, physical therapy specialty, rehabilitation

## Abstract

Background: The impact of muscle-training treatment on quality of life and functional outcomes in people with pelvic floor dysfunction may be related to adherence rates. Methods: Nine electronic databases were searched for studies published up to 15 October 2024. A qualitative synthesis was used to describe the relationship between adherence or compliance with treatment, quality of life, and symptomatic severity. A meta-analysis of data from selected studies was performed that assessed quality of life and symptomatic severity in the short term. Results: Seven studies with 2190 participants were included. Of these studies, 42% showed rates greater than 80% in terms of adherence. A beneficial effect was found in terms of urinary incontinence severity without statistical differences between the groups (*p* = 0.813), while quality of life showed statistically significant improvements favoring the experimental group (*p* = 0.036). The quality of the evidence was collected or measured from low to high. Conclusions: People with pelvic floor disorders show high rates of adherence to pelvic floor muscle exercise and experience an improved quality of life in the short term, but more research is needed on the design of homogeneous systems to measure compliance and adherence to exercise-based treatments.

## 1. Introduction

The pelvic floor, composed of muscular tissue, fibromuscular, and fascial elements, extends from the pubis to the coccyx and contains several layers. The middle layer of the pelvic floor is made up predominantly of muscle tissue, which, through contraction and relaxation, supports the pelvic organs [1]. It plays an important role in urinary and fecal continence and sexual and reproductive function and is essential for good health and well-being [2,3]. However, its normal function can be altered by increasing or decreasing muscle tone, pain due to impaired muscle coordination, or involvement of the pudendal nerve [4]. In women, the disorders of pelvic floor muscle are related to pregnancy and childbirth [5] or menopause [6]. The general prevalence is 46%, but the most common condition is urinary incontinence (hereafter UI), affecting 30–60% of individuals [7]. In men, diagnoses are classified [8] as storage disorders, voiding dysfunction, and mixed storage and voiding dysfunction, reflecting lower urinary tract function and related to sexual health impairment [9]. The risk factors for UI in men include older age, lower urinary tract symptoms, infections, functional and cognitive impairment, neurological disorders, and prostatectomy [10]. People with pelvic floor disorders develop a disability (a symptom of pelvic floor dysfunction) that has a negative impact on their activity or participation (quality of life) [11,12,13]. Sexual health affects not only people with pelvic floor dysfunctions in their lives but also that of their partners [14,15].

The therapeutic approach to pelvic floor disorders is commonly multidisciplinary. Pelvic floor muscle training (hereafter PFMT) is a therapy to improve endurance, strength, power, relaxation, or a combination of these, and because of its effectiveness is considered the first line of treatment for stress UI in women [2,6,16,17], and in men [18]. Thus, PFMT, by improving muscle function, has been reported to ameliorate patient disability, activity, and participation [12], which leads to improved psychosocial outcomes [19,20]. Exercise, in general and PFMT in particular, seems to be positive in sexual function in males after prostate cancer, but further research would be necessary to determine appropriate exercise interventions [21].

In both male and female populations, the effectiveness of treatments based on exercise depends on several factors, including maintenance of the routine over time, for which the concept of compliance and adherence to treatment is fundamental [22,23].

The National Library of Medicine [24] defines compliance and adherence to treatment as “The extent to which the patient follows the prescribed treatment, such as keeping appointments and schedules and adherence to medication for the desired therapeutic outcome”. Numerous research studies have shown the importance of adherence [25,26] and compliance [27,28] to treatment in general and to exercise [29] in particular [30,31,32,33]. We also know the complexity derived from the high number and heterogeneity of the barriers and facilitators of adherence to treatment and its multidimensional condition [34,35,36]. The most suitable objective and homogeneous measurement instrument for this concept is the questionnaire [33,37]. The scientific community agrees on two important aspects of adherence: that it tends to decrease over time, especially in the long term [38], and that it is considered to be high when it is above 80% [29,39,40,41,42]. 

It seems that the importance of adherence and compliance lies in the relationship with the results obtained after applying a given treatment. A high adherence rate is assumed to indicate that the outcome of the intervention derives from it. For this reason, there are studies that only collect data from those patients who have completed treatment as indicated.

Nagpal et al. [29] offered a possible way to analyze the relationship between adherence to exercise-based treatments and therapeutic effectiveness, showing us four possible scenarios: no significant effect of the intervention or improvement is observed compared with the inactive control group related to low (Scenario 1) or high (Scenario 2) adherence; intervention favors the experimental group over the control group related to low (Scenario 3) or high (Scenario 4) adherence. These scenarios give us the opportunity to interpret the results of clinical trials in a very interesting way. 

Therefore, the objective of this systematic review was to analyze the effectiveness of exercise-based treatments on quality of life and severity of pelvic floor dysfunction and the relationship between compliance or adherence to exercise-based treatments and therapeutic effectiveness. 

## 2. Materials and Methods

This systematic review was registered (14 October 2024) in PROSPERO (CRD42022349487) and was conducted according to the PRISMA [43] guidelines, and no modifications were made to the initial protocol during this study.

### 2.1. Data Sources and Searches

A systematic search was conducted independently by two researchers in PubMed, CINAHL, Medes, Web of Science, EMBASE, Scopus, PEDro, The Cochrane Library, and TRIP databases to identify all studies published related to the topic up to 15 October 2024. To minimize publication bias, a search was conducted in the gray literature and clinical trial registry databases. The search strategy detailed in Table A1 was applied, along with specific strategies tailored to each database (Table A2). The search was limited to randomized clinical trials published in Spanish and English without restrictions on the time of publication.

### 2.2. Study Selection 

This process, according to the eligibility criteria, was carried out independently by three researchers as follows: the titles and abstracts were screened; from the studies that met the eligibility criteria, the full texts were recovered and screened. Those studies in which the eligibility criterion “Studies that measure, in addition to effectiveness in terms of symptom improvement, adherence to treatment collected through specific scales or questionnaires” did not appear clearly collected in the abstract were selected full texts to avoid potential losses. The study selection process is described in detail in Table A3.

For collection, filtering, removal of duplicates, and citation and reference management, Zotero v. 6.0.36 software was used.

#### Eligibility Criteria

Randomized clinical trials (RCT) were selected for inclusion in this study. Participants included patients of both sexes diagnosed with pelvic floor disorders of non-neurological origin. The intervention reviewed was physiotherapy treatment focused on PFMT. As comparators, studies that evaluated other interventions, such as medication or surgery, as well as those in which participants did not receive physiotherapy treatment for pelvic floor dysfunction, were included. Finally, only studies that assessed both symptom improvement and adherence to treatment using specific scales or questionnaires were considered.

### 2.3. Outcome Measures

#### 2.3.1. Main Outcome

Compliance and adherence to exercise-based physiotherapy for pelvic floor disorders in the short and medium term, collected through specific questionnaires or scales, measured in terms of days of exercise practice, number of exercises performed, or frequency of attendance to physiotherapy sessions.

#### 2.3.2. Secondary Outcomes

Quality of life is a generic concept reflecting concern for the modification and improvement of attributes of life, physical, political, moral, and social environment, as well as health and illness, measured through valid and reliable questionnaires or scales. The severity of pelvic floor dysfunction as levels within a diagnostic group that are established by various measurement criteria applied to the seriousness of a patient’s disorder (urine or fecal loss, leakage episodes, and erectile dysfunction), measured through specific test or questionnaires (pad test and a bladder diary). 

### 2.4. Data Extraction and Quality Assessment

Data extraction was carried out independently by two researchers using the full texts of the selected studies. Data related to a study’s participants, intervention, and results according to the main and secondary variables were extracted and collected in tables for analysis. Author/year and country from studies. Information regarding participants (pelvic floor disorder, sample size, and sex). Intervention and outcome variables (exercise protocol, control group intervention, strategy to improve adherence to treatment, outcome measures and measurement instruments, participants who complete the intervention period, and losses in the follow-up).

Methodological quality was assessed using the PEDro Scale [44] by two independent reviewers with the intervention of a third in the event of discrepancies. The PEDro scale allows the identification of randomized clinical trials for external validity (item 1) and internal validity (items 2–9) and which may have sufficient statistical information to make their results interpretable (items 10–11). The scale is measured out of 10 since criterion 1 is not used for the calculation of the score [45].

### 2.5. Effects Measures 

Compliance and adherence: Frequency in terms of attending physiotherapy sessions, days of exercise practice, number of exercises performed, and their means. Quality of life: questionnaire scores in terms of frequency (mean differences). Severity (of pelvic floor dysfunction): frequency (mean differences), relative risks.

### 2.6. Data Synthesis and Analysis

Data were analyzed using a qualitative synthesis and, whenever possible, a quantitative synthesis (meta-analysis). Standardized mean differences (SMD), estimated effect, and standard error, with 95% confidence intervals (CI) were calculated. Fixed or random effects models were used according to the degree of heterogeneity, using the I2 coefficient. Specifically, for I^2^ > 50% and *p* < 0.05, which indicates substantial heterogeneity, random effects models were used, and when I^2^ < 50% and *p* > 0.05, which indicates substantial homogeneity, fixed effect models were used. The Jamovi version 2.3.28 software was used to summarize the effects and build the forest plots. Egger’s regression test was used to estimate publication bias.

The approach proposed by the Grading of Recommendations Assessment, Development, and Evaluations (GRADE) [46], which allowed to classify the evidence as high, moderate, low, or very low and to discern the importance of the results, was used.

## 3. Results

### 3.1. Flow of Trials Through the Review

**Figure 1:**
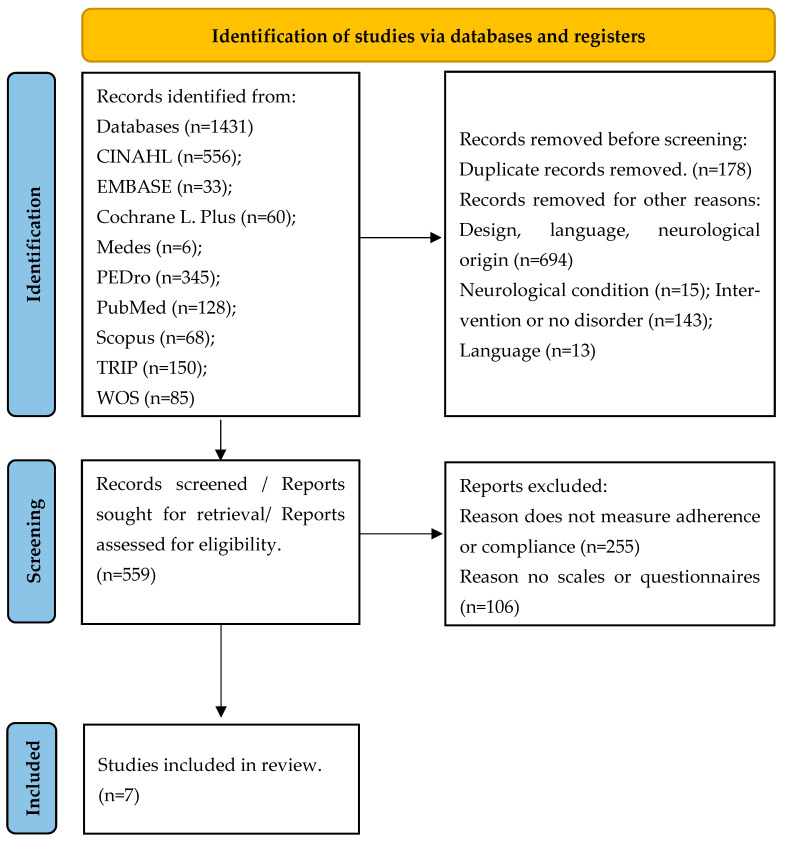
PRISMA 2020 flow diagram.

A total of 1431 articles were identified. Following the recommendations of PRISMA [43], after eliminating duplicates and those that did not meet the eligibility criteria after reading the title and abstract, the full texts of 559 studies were selected (see Table A3). Finally, seven studies were included in the selection, as shown in Figure 1.

### 3.2. Characteristics of the Included Studies 

The main characteristics of the included studies [47,48,49,50,51,52,53] are shown in detail in Table 1. The included studies reported data for 2190 participants. Six trials reported data from 1337 women (61%) [48,49,50,51,52,53] and one from 853 men (39%) [47]. The study conducted in men addressed UI related to radical prostatectomy (hereafter RP) or transurethral prostate resection (hereafter TUPR). Both are surgical interventions performed in patients with prostate cancer (RP) or benign prostatic hyperplasia (TUPR) [47]. Of the investigations conducted in women, two excluded pregnant women [52,53], and four included pregnant [49,51] or postpartum women [48,50,51].

All clinical trials studied UI. One RCT included fecal incontinence [48]. None of the studies exclusively included fecal incontinence, pelvic organ prolapse, or another pelvic floor disorder. One work does not specify the PFMT protocol [51]. 85% of the protocols are carried out daily: once a day [48,52,53], twice [47,50], or three times a day [49]. Dosage varies widely between studies. Detailed protocols for each intervention can be found in Table 2. All investigations in the sample collected results on the efficacy of PFMT-based physical therapy treatment, quality of life, and compliance or adherence to treatment at some point during the study. The intervention period ranged from a single intervention to 4 months. Three studies did not follow up [49,52,53]. The remaining ranged from 1.5 months to 12 months (see Table 1). The percentages of loss to follow-up, as can be seen in detail in Table 3, ranged from 5% [48] to 87% [51]. One study found losses of less than 15% [47]. The difference between losses was similar between the experimental and control groups in four works [47,51,52,53]. In the rest of the studies, this difference was higher [49] or slightly higher in the control group [48,50].

### 3.3. Quality Assessment 

The results of the risk of bias assessment of the included studies using the PEDro Scale are shown in Table 4. All studies in the sample met criteria 1–4, 10, and 11. One study (14%) blinded participants [51]. No study blinded therapists. One study (14%) blinded the assessors [49]. One study (14%) met criterion 8 (adequate follow-up) [47]. Intention to treat was not applied in two studies (28%) [52,53].

### 3.4. Main Outcome 

The results in terms of compliance and adherence to treatment of the included studies are shown in detail in Table 5.

Compliance and/or adherence to treatment were measured in the short (85%) [47,49,50,51,52,53], medium (14%) [47], or long-term (28%) [47,48]. During the intervention period, 71% [47,49,51,52,53] measured compliance or adherence to treatment. In the follow-up period, 57% showed results [47,48,50,51].

Different questionnaires were used across studies to measure adherence, including the Knowledge Attitude Practice questionnaire [51], the MAPS questionnaire [47], the Exercise adherence rate scale (EARS) [49], visual analog scales [52,53], or questionnaires used in previous studies [48,50].

One study examined whether participants forgot to exercise [49], and one specifically studied barriers to treatment specifically, including forgetfulness [50].

All works showed strategies to improve compliance and adherence, which were based on contact with clinicians by telephone or face-to-face contact [47,49,51], information leaflet [47,50,53], and reminders in paper, video, or APP format [47,49,51]. These strategies were carried out in the experimental group except in two studies where visits to the clinic for electrostimulation treatment without PFMT [52] or written bladder training instructions were carried out [53] in the control group. 

Among the seven included studies, the practice or non-practice of treatment, the frequency [47,49,51,52], the quantity [47,48] (amount of exercises or time, number of repetitions, sets, or sessions) [48,49,50], and perceived compliance with treatment were measured [52,53]. Of these, two showed adherence rates lower than 80% [47,48] (the study by Glazener et al. from 2001 [48] showed 78% of adherence. In surveys conducted at 6 and 12 years [56], the authors observed that adherence had declined to match the control group). 

Three RCTs (42%) showed rates greater than 80% [50,52,53]. Two studies showed minimal or significant improvement in adherence but did not show whether it was greater or lower than 80% [49,51]. All studies that showed high adherence measured it in the short term [50,52,53]. The two investigations that showed low adherence measured it in the long term [47,48].

### 3.5. Secondary Outcomes

The results in terms of the severity of pelvic floor disorders and the quality of life and its relation to sexual health outcomes of the included studies are shown in detail in Table 6.

Six studies (85%) measured both severity of symptoms and quality of life in the short (1–3 months) [47,49,50,51,52,53], one (14%) in the medium (6–9 months) [47], and two (28%) in the long term [47,48].

To collect results on symptomatic severity, the ICIQ-Urinary Incontinence Short Form (ICIQ-UI-SF) [47,49,50,51], The Incontinence Severity Index (ISI) [52,53] and a UI frequency question [48] were used as instruments.

Quality of life was measured by ICIQ-UI-SF (third question) [47,50,51], European Quality of Life-5 Dimensions EQ-5D [47], Short Form questionnaire-12 items SF-12 [47], ICIQ-Lower Urinary Tract Symptoms quality of life (LUTSqol) questionnaires ICIQ-LUTSqol [49], The Kings Health Questionnaire (KHQ) [52], Urogenital Distress Inventory, and Short Form UDI-6 with Incontinence impact questionnaire short form IIQ-7 [53]. One study used the Hospital Anxiety and Depression Scale HADS [48]. 

Of the eight questionnaires used, two measure sexual health directly: ICIQ-Lower Urinary Tract Symptoms quality of life (LUTSqol) questionnaires ICIQ-LUTSqol [49] directly measures how incontinence affects sex life. The Kings Health Questionnaire (KHQ) [52] directly measures the impact of incontinence on sexual health. 

The remainder measured it indirectly: ICIQ-UI-SF (third question) [47,50,51], indirectly related to sexual health (impact of incontinence). European Quality of Life-5 Dimensions EQ-5D [47], indirectly related to anxiety and pain affect sexuality. Short Form questionnaire-12 items SF-12 [47], indirectly related (as physical and emotional well-being affect sexuality). Urogenital Distress Inventory, Short Form UDI-6 [53], indirectly related (as urogenital complaints affect sexuality). Incontinence impact questionnaire short form IIQ-7 [53], indirectly related (incontinence affects sex life). The Hospital Anxiety and Depression Scale HADS [48] is indirectly related (anxiety/depression affects sex life). 

In 86% of the included studies [48,49,50,51,52,53], changes in symptomatic severity were related to changes in the quality of life. There was an improvement in symptomatic severity in 71% of studies [48,50,51,52,53], and 86% showed an improvement in the quality of life [47,48,50,51,52,53]. Symptomatic severity did not improve in the study in men during the intervention period or at follow-up [47]. In this trial, the quality of life improved at follow-up but because of recovery from surgery. 

Three studies (42%) showed significant statistical differences in favor of the experimental group in both severity of symptoms and quality of life [48,51,53]. The rest did not show differences between the groups in any variable [47,49,50,52]. No study showed better results in the control group. 

### 3.6. Relationship Between Sexual Health and Secondary Outcomes 

Sexual health was measured directly through symptomatic severity in one study [47] and through quality of life in two studies [49,52]. In the remaining studies in the sample, sexual health was measured indirectly, as shown in Table 6. 

A total of 71% of the sample studies [48,50,51,52,53] showed improved sexual health, as measured by symptomatic severity or quality of life. Of these studies, the improvement was in favor of the experimental group in 42% of the sample [48,51,53].

### 3.7. Relationship Between the Main and Secondary Outcomes

From the studies in the sample and in accordance with the scenarios proposed by Nagpal et al. [29], this review found the following results (Table 7). One study was related to Scenario 1 (no significant effect of intervention or improvement is observed in comparison with the inactive control group related to low adherence) [47]. Two were related to Scenario 2 (no significant effect of intervention or improvement is observed in comparison with the inactive control group related to high adherence) [50,52]. Scenario 3 was related to a study (intervention favors the experimental group over the control group related to low adherence) [48]. Finally, one study was related to Scenario 4 (intervention favors the experimental group over the control group related to high adherence) [53]. This review could not analyze this issue in two studies because of the lack of information in terms of adherence percentages. Both showed minimal or significant improvement in adherence but did not show whether it was greater or lower than 80% [49,51].

In relation to treatment adherence rates, these were high in three [50,52,53] of the five studies [48,50,51,52,53] in which patients’ sexual health (through improvement in symptomatic severity or quality of life) improved but only in one study with significant differences between groups [53].

### 3.8. Results of the Quantitative Syntheses

**Figure 2:**
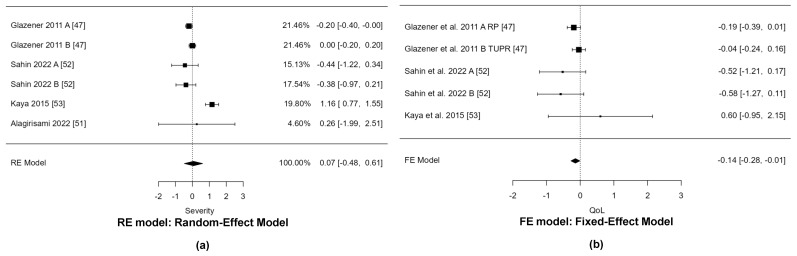
(**a**) Forest plot of the meta-analyses of disability (Urinary incontinence severity); (**b**) Forest plot of the meta-analyses of activity or participation (quality of life) in the short term.

Figure 2 shows the forest plot of the meta-analysis of the selected studies that evaluated the severity of symptoms and quality of life in the short term. There were no statistically significant differences between the groups (SMD = 0.07; 95% CI [−0.48, 0.61], *p* = 0.813) in terms of the severity of symptoms. There was a statistically significant difference in favor of the experimental group (SMD = −0.12; 95% CI [−0.28, −0.01], *p* = 0.036) in terms of quality of life. The timing and instruments for collecting results and meta-analyses can be consulted in Table A4.

### 3.9. Publication Bias Assessment

The Egger’s test did not reveal publication bias (*p* > 0.05). Figures S1 and S2 in Supplementary Materials show the funnel plots for the meta-analyses.

### 3.10. Evidence Synthesis

The evidence synthesis carried out using GRADEpro v. 3.0 tool considered severity, quality of life, compliance, and adherence to treatment in Table S1. All results were classified as “not important”. The level of certainty in treatment and the outcome of adherence was low. There was a high level of certainty in the meta-analysis performed with respect to the quality of life and moderate severity of symptoms.

## 4. Discussion

The purpose of this systematic review was to analyze the adherence to exercise-based treatments in patients with pelvic floor disorders, given its importance in interpreting the effectiveness of exercise intervention studies [29]. In a recent scoping review [57] aimed at reviewing research priorities in physiotherapy, nine categories of research priorities were identified. Of these, the study of adherence appears as a line of research in two of them (patient needs, expectations, experience, and context; technology and big data). This review included both the male and female populations in accordance with reference studies on adherence carried out in patients with other pathologies [25,26,27,28]. However, the sample had a larger number of studies conducted in women than in men; only one study [47] carried out in the male population met the eligibility criteria.

Most of the sample was conducted in a population with urinary incontinence, so there appears to be a lack of studies that measure the adherence to PFMT in populations with other pelvic floor dysfunctions.

### 4.1. Qualitative Analysis

Qualitative analysis of the included studies revealed that adherence and compliance with treatment appeared to be high in the short term and decreased in the long term. This fact is well known and therefore, recommendations for improvements in the lines of research [38] are based on efforts to maintain adherence rates in the medium and long term. In this review, the studies agreed on the response criteria, characterizing adherence as high when it exceeded 80%. This is in line with previous publications submitted between 1996 and 2023 [29,39,40,41,42]. Although data show that high short-term adherence predicts high long-term adherence, rates are also known to decline over time [29,38]. This review found that studies tend to measure outcomes in the short term and a deficit in the medium and long term. The review studies used strategies based on contact with the therapist, reinforcement, and reminders. This is because forgetfulness is known to be a major barrier, and increased contact with the therapist is a relevant facilitator [25,27]. However, despite these strategies, this review found studies that did not show high adherence rates.

Sexual health was measured indirectly in most of the studies in the sample through the severity and/or quality of life of the patients. In the qualitative analysis, the severity of symptoms appeared to be related to the quality of life. There was an improvement in symptomatic severity in 71% of studies [48,50,51,52,53], and 86% showed an improvement in the quality of life [47,48,50,51,52,53]. With these data, this review attempted to correlate treatment effectiveness with adherence and compliance rates using the scenarios proposed by evidence [29]. In the sample, the studies were related to Scenarios 1 [47], 2 [50,52], 3 [48], and 4 [53].

However, these results should be viewed with caution. As could be analyzed with the GRADEpro v. 3.0 tool, the level of certainty in treatment and adherence outcomes was low. Heterogeneity in the PFMT protocols and adherence measurement systems makes it difficult to interpret the results correctly. In the particular case of the pelvic floor pathology approach, due to the difficulty of blinding subjects and therapists, scores of 8 can be considered high quality and between 4 and 6 moderate quality according to the PEDro scale [44]. All studies included in the sample are of moderate quality according to the PEDro scale and meet the 11 criteria (this study provides both point measures and measures of variability for at least one key outcome). However, when the statistical information comes from adherence and/or compliance, it appears heterogeneous.

This review did not find a clear relationship between improvements in symptomatic severity or quality of life (and sexual health) and adherence rates to exercise-based treatment. In fact, of the studies in which the experimental group showed improvements compared with the control group, the adherence rates ranged from high to low. It seems reasonable to think that if adherence is high, the effectiveness of the treatments will be higher. However, Nagpal et al. [29] offered a possible way to analyze the relationship between adherence to exercise-based treatments and therapeutic effectiveness, showing four possible scenarios. In the sample, one study showed low adherence and low effectiveness [47]. This study, which falls into Scenario 1, suggests that we should strive to increase adherence to see if we could achieve better results in terms of effectiveness. However, this review also found studies in which high adherence did not lead to better outcomes in the experimental group compared with the control group. In fact, all four possible scenarios proposed in the study by Nagpal et al. [29] were met; therefore, a clear relationship between the variables could not be established.

### 4.2. Quantitative Analysis

The quantitative analysis of the results revealed that PFMT seems to be associated with improvements in the global quality of life in the short term. There were no statistically significant differences in terms of the severity of symptoms related to urinary incontinence. Therefore, a quantitative analysis could not be performed to interpret these data in detail, nor could a detailed study of the relationship between exercise adherence and exercise efficacy be carried out. It seems that the main drawback lies in the lack of homogeneity in the way the results of this variable are measured. Nevertheless, this is not the case for instruments that measure the quality of life or symptomatic severity. Moreover, this review found questionnaires (i.e., ICIQ-UI-SF) that were commonly used in a diverse population, were easy to use, and allowed us to carry out a more rigorous analysis of the results.

### 4.3. The Challenge of Adherence

During the screening process, 255 studies were eliminated because they did not measure adherence to treatment. A total of 106 were eliminated because they measured compliance or adherence through the patient’s diary, reports made verbally, or through information approved by clinicians. Furthermore, this review detected more than 15 studies that specifically stated in their limitations that they did not measure adherence. From some of these, lines of research on the measurement of adherence were created in 1998 [58] and 2006 [59]. However, the rest of the studies with this limitation were subsequently published; eight were published in the last five years, including 2024 [60]. This is echoed in similar reviews conducted recently in male [23] and female [61] populations in which this outcome appeared as secondary. Although they had different samples, all three studies agreed on the problem of measuring adherence to treatment. Therefore, this seems to be the first meta-analysis carried out in people with pelvic floor disorders that highlights the lack of importance given to adherence and compliance to treatment in trials of the effectiveness of pelvic floor muscle exercise treatment, despite the relevance given to these variables by the scientific community. Aware of the multidimensionality of adherence and although there are questionnaires that take it into account [33,55], perhaps the key lies in the use or design of a general questionnaire on adherence and compliance, from which, as in other cases, such measurement of quality of life, specific questionnaires can be derived. In this way, in addition to homogenizing the results and being able to perform a quantitative synthesis, we will address an additional problem. If data obtained on adherence are based only on participants who completed the questionnaires, we are losing valuable information [29].

### 4.4. Clinical Implications 

For future research and clinical practice in the pelvic floor disorder area, we may need to strive for high rates of compliance with questionnaires or scale submissions, just as we strive for high rates of adherence to treatment. Until we can adequately relate exercise adherence to exercise effectiveness, we will not be able to address the disability of people suffering from pelvic floor dysfunction. If we do not measure adherence and compliance with treatment of people with pelvic floor dysfunctions with tools such as those used to measure variables such as quality of life, we will never really know the clinical effectiveness of treatments. 

### 4.5. Limitations and Future Considerations

This systematic review has some limitations. The sample is small, but this is perhaps one of the most interesting results because it is related to the lack of available evidence to measure adherence and compliance through objective systems. Likewise, the search restricted to studies in English and Spanish could have generated a language bias, potentially excluding evidence published in other languages. The lack of significance in Egger’s test does not completely rule out the possibility of publication bias due to the small sample size. A meta-analysis of the main outcome could not be performed because of the variability in the measurement instruments and statistical data provided by the studies. Finally, limited clinical implications can be drawn from the meta-analyses. The results of the meta-analysis of symptomatic severity have a confidence interval that ranges from a moderately harmful effect to a moderately helpful effect, so pooled data do not narrow down the possible effect enough to draw any coherent clinical implication from this imprecise estimate. The results of the quality of life meta-analysis had a confidence interval ranging from a small to moderate effect to a trivial effect (−0.01), so we redetermined whether the effect of the intervention was clinically relevant or clinically insignificant.

## 5. Conclusions

The results appear to indicate, with a low level of certainty of evidence, that people with pelvic floor disorders show high rates of adherence to pelvic floor muscle exercise in the short term and experience an improved quality of life with a high level of certainty of evidence. However, a robust relationship between adherence rates and intervention efficacy cannot be established. More randomized clinical trials are required to support these findings.

Therefore, more research is needed on the design and implementation of a scientifically recommended and easy-to-administer system to measure compliance and adherence to exercise-based treatments. This would allow the establishment of a clear relationship between compliance or adherence data and the efficacy of treatment, quality of life, or disability caused by pelvic floor disorders.

## Figures and Tables

**Table 1:** Descriptive characteristics of the included studies.

Author/ Year/ Country	*n*/Participants/ Dysfunction	Intervention/ Follow Up	Outcomes Measures/Instruments
Glazener et al. (2011) [47] UK	*n* = 853 Men UI (SUI, MUI, UUI) 6 wk after RP prostate cancer or TURP in the case of BPH	3 mo. EG: PFMT. Bladder training if IUU. CG: Standard care (non-expert advice on PFMT.) Follow up: 12 mo.	MO: subjective report of urinary continence at 12 mo. Primary cost-effectiveness measure: QALYs. SO: clinical outcomes: urinary outcomes (presence, frequency, and severity of incontinence, effect of incontinence on quality of life, use of pads and catheters, type of incontinence, urinary frequency, and nocturia); bowel outcomes (fecal incontinence, constipation, bowel urgency); sexual function (erectile function, ejaculation, change in sexual function) QoL, health service use for UI, other health service use, effects of interventions (PFMT use and lifestyle changes), and economic measures. ADH, Satisfaction with treatment. MAPS Questionnaire: ICI-SF/10-point scale ICI-Q/Short Form questionnaire-12 items (SF-12)/European Quality of Life-5 Dimensions (EQ-5D)/3 days urinary diary QALYs derived from responses to the EQ-5D and SF-12; and the effect of the intervention in changing health-related behavior and practice of PFMT and bladder training or urge suppression.
Glazener et al. (2001) [48] UK/New Zealand	*n* = 747 Women SUI/ UUI /MUI /FI persistent 3 months postpartum	1 visit with reviews at 7 y 9 mo. EG: PFMT (5 mo. postpartum), bladder training, if necessary, at visit 7/9 mo. CG: peripartum preparation such as EG, (sometimes included PFMT) Follow up: 12 mo. EG: home-based PFMT/CG: nothing	Does not assess in the intervention. Follow up: MO: persistence and severity of UI 12 mo. postpartum. SO: PFMT performance, change in coexisting FI, well-being, and overall UI severity rating. Anxiety and depression. Other: PFMT use and frequency and pad use. UI Severity (at least 1 time/wk). FI Severity. HADS/ADH: questionnaire.
Jaffar et al. (2022) [49] Malaysia	*n* = 26 pregnant women >18 yr with SUI/MUI	2 mo. EG: 8 wk behavioral change intervention PFMT KEPT app. (mHealth) GC: regular antenatal care. (APP after 8 wk). No follow-up	MO: PFMT ADH SO: UI, Symptomatic severity of UI, QoL, knowledge, attitude, practice, and self-efficacy of PFMT. (EARS)/ICIQ-UI SF/ICIQ-LUTSqol/validated scale/SESPPFE
Sacomori et al. (2020) [50] Brazil	*n* = 202 women > 18 yr postpartum period with a living child SUI	1 individual postpartum session. EG: home-based PFMT during breastfeeding. CG: Routine postpartum hospital care (no PFMT) Follow up: 3 mo. EG: Unsupervised home-based PFMT/CG: none.	EG/CG Urinary symptoms before pregnancy/3rd trimester/3rd month postpartum after 1 session of PFMT training (ITT). QoL baseline/3º trimester. Follow up: (in abstract it appears as a primary measure QoL). MO: ADH (time spent exercising) and barriers to treatment. (EG) SO: Involuntary loss of urine and QoL Questionnaire/ICIQ-SF
Alagirisamy et al. (2022) [51] Malaysia	*n* = 170 women pregnancy- Postpartum UI	Before 22 wk gestation and up to 38 wk gestation. EG: PFMT + perinatal care. CG: Perinatal, ante, and postnatal hospital care (includes info UI/PFMT). Follow-up: 4–6 wk after delivery.	MO: knowledge, attitude, practice, and self-efficacy of PFMT. SO: Continence and UI Severity EG/CG at baseline and at 3 time points: 1T (28–30 wk gestation), 2T (36–38 wk gestation), and 3T (4–6 wk postpartum). KAP Q/SESPPFE/ICIQ-UI SF
Sahin et al. (2022) [52] Turkey	*n* = 60 Women UI SUI	2 mo. Home-based PFMT. G1: EES G2: PFMT G3: EES + PFMT No follow-up.	Self-reported improvement/UI severity/symptomatic distress/QoL/UI episodes/strength, endurance/dysfunction ADH. 4 items Likert scale/KHQ/pad test/ISI/24 h urinary dairy/perineometry/PFDI-20. ADH: VAS scale
Kaya et al. (2015) [53] Turkey	*n* = 132 Women SUI UUI MUI	6 wk. BT: bladder training. EG: BT + PFMT CG: BT No follow-up.	MO: Self-reported improvement SO: UI severity/symptomatic distress/QoL/average nº of UI episodes and urination per day/strength (PFMS) endurance (PFME). 4 items Likert scale/ISI/UDI-6/IIQ-7/3 24-h frequency-volume graph on three different days/diaries/perineometry. ADH: VAS

UI: Urinary incontinence; SUI: Stress Urinary Incontinence; UUI: Urge Urinary Incontinence; MUI: Mixed Urinary Incontinence; FI: Fecal incontinence; EG: experimental group; CG: control group; PFMT: Pelvic floor muscle training; ADH: treatment adherence; QoL: Quality of life; RP: Radical Prostatectomy; TUPR: transurethral resection of the prostate; BPH: Benign prostatic hyperplasia; QALY: incremental cost per quality-adjusted life year; ICIQ-SF: International Consultation on Incontinence Questionnaire; ICIQ-UI-SF: ICIQ-Urinary Incontinence Short Form; EQ-5D European Quality of Life-5 Dimensions; SF-12 Short Form questionnaire-12 items; HADS: Hospital Anxiety and Depression Scale; EARS: Exercise Adherence Rating Scale; ICIQ-LUTSqol: ICIQ-Lower Urinary Tract Symptoms quality of life (LUTSqol) questionnaires; SESPPFE: Self-Efficacy Scale for Practicing Pelvic Floor Exercise Questionnaire; KHQ: The Kings Health Questionnaire; KAP Q: Knowledge Attitude Practice Questionnaire; ISI: The Incontinence Severity Index; PFDI-20: Pelvic Floor Distress Inventory-20; VAS: visual analogue scale; UDI-6: Urogenital Distress Inventory, Short Form; IIQ-7: Incontinence Impact Questionnaire; G1: Group 1; G2: Group 2; G3: Group 3; MO: main outcome; SO: secondary outcome; EES: External Electro Stimulation.

**Table 2:** Pelvic Floor Muscle Training protocols.

Study	Length	Freq. (Times/d)	Dosage
Glazener et al. (2011) [47]	3 mo. F.U: 12 mo.	Daily (×2)	EG 3 maximum con. Up to 10 s/10 s rest. Decubitus, Sitting, and standing.
Glazener et al. (2001) [48]	Single session F.U:12 mo.	Daily	EG 8 to 10 sets of fast and slow con. Until 80–100 repetitions are reached.
Jaffar et al. (2022) [49]	2 mo.	Daily (×3)	EG Beginner: 2 s con./Intermediate: 6 s con./Expert: 10 s. con./6 s rest × 10 rep.
Sacomori et al. (2020) [50]	Single intervention F.U:3 mo.	Daily (×2)	EG 10 reps. of 10 s of maximum intensity/10 rep. of 1 s.
Alagirisamy et al. (2022) [51]	4 mo. F.U:1.5 mo.	No information.	No information.
Sahin et al. (2022) [52]	2 mo.	Daily	(G2/G3) Home 1 set 10 fast 10 slow con. (5 s, hold 10 s/5 s). 2 1st week, 2 sets/d (20 fast and 20 slow). Every 2 weeks, 2 more sets were added, 8 sets (80 fast and 80 slow con./d at the end of 8th week. Sitting, supine, semi-squatting, and standing.
Kaya et al. (2015) [53]	1.5 mo.	Daily	EG Home-based. Fast (2 s) and slow.1 slow con. 15 s (5 s con./5 s maintenance/5 s relaxation). 1 set 10 fast/10 slow. 1st wk 5 sets/d (5 × 10 fast and 10 slow) progressively >5 sets/wk: 30 in the 6th wk daily (300/300). Supine, sitting and standing.

F.U: Follow-up; rep.: repetitions; con.: contractions; G2: Group 2; G3: Group 3.

**Table 3:** Losses in the follow-up period.

Study	Randomized (*n*)	Participants Completed the Treatment Period-Dropped Out	% of Follow-Up Losses (Time Elapsed)
Glazener et al. (2011) [47]	RP *n* = 411 (EG = 205, CG =206)	RP *n* = 391 (EG = 196, CG = 195) RP *n* = 20 (EG = 9, CG = 11)	Total: 5% (12 mo.) RP: EG 4%, CG 5%
	TUPR *n* = 442 (EG = 220, CG = 222)	TUPR *n* = 397 (EG = 194, CG = 203) TUPR *n* = 45 (EG = 26, CG = 19)	Total 10% (12 mo.) TUPR: EG 12%, CG 8.5%
Glazener et al. (2001) [48]	*n* = 747 (EG = 371, CG = 376)	*n* = 692 (EG = 371; [Received intervention as allocated = 316 ITT], CG = 376) *n* = 223 (EG = 92, CG = 131)	Total: 30% (12 mo.) EG 25%, CG 35%
Jaffar et al. (2022) [49]	*n* = 26 (EG = 16, CG = 10)	*n* = 16 (EG = 10; [ITT = 13], CG = 6 [ITT = 10]) *n* = 12 (EG = 6, CG = 6)	Total: 46% (2 mo.) EG 37.5%, CG 60%
Sacomori et al. (2020) [50]	*n* = 202 (EG = 98, CG = 104)	*n* = 132 (EG = 67, CG = 65) *n* = 70 (EG = 31, CG = 39)	Total: 35% (3 mo.) EG 31%, CG 37.5%
Alagirisamy et al. (2022) [51]	*n* = 170 (EG = 85, CG = 85) ITT Modified analysis (EG = 65, CG = 59)	*n* = 21 (EG = 10, CG = 11) *n* = 149 (EG = 75, CG = 74)	Total: 87% (3 mo.) EG 88%, CG 87%
Sahin et al. (2022) [52]	*n* = 60 (G1 = 20, G2 = 20, G3 = 20)	*n* = 51 (G1 = 17, G2 = 17, G3 = 17) *n* = 9 (G1 = 3, G2 = 3, G3 = 3)	(No follow-up) Total: 15% EG 15%, CG 15% complete the intervention.
Kaya et al. (2015) [53]	*n* = 132 (EG = 67, CG = 65)	*n* = 108 (EG = 56, CG = 52) *n* = 24 (EG = 11, CG = 13)	(No follow up) Total: 18% EG 16%, CG 20%

EG: experimental group; CG: control group; RP: radical prostatectomy; TUPR: transurethral resection of the prostate; G1: Group 1; G2: Group 2; G3: Group 3.

**Table 4:** Methodological classification of quality according to the PEDro Scale.

Study	1	2	3	4	5	6	7	8	9	10	11	Total
Glazener et al. (2011) [47]	Y	Y	Y	Y	N	N	N	Y	Y	Y	Y	7
Glazener et al. (2001) [48]	Y	Y	Y	Y	N	N	N	N	Y	Y	Y	6
Jaffar et al. (2022) [49]	Y	Y	Y	Y	N	N	Y	N	Y	Y	Y	7
Sacomori et al. (2020) [50]	Y	Y	Y	Y	N	N	N	N	Y	Y	Y	6
Alagirisamy et al. (2022) [51]	Y	Y	Y	Y	Y	N	N	N	Y	Y	Y	7
Sahin et al. (2022) [52]	Y	Y	Y	Y	N	N	N	N	N	Y	Y	5
Kaya et al. (2015) [53]	Y	Y	Y	Y	N	N	N	N	N	Y	Y	5

1. Eligibility criteria; 2. Random allocation; 3 Concealed allocations; 4. Baseline comparability; 5. Blind subjects; 6. Blind therapists; 7. Blind assessors; 8. Adequate follow-up; 9. Intention-to-treat analysis; 10. Between-group comparisons; 11. Point estimates and variability; Y, yes; N, no.

**Table 5:** Results Main outcome: compliance and adherence to treatment.

Study	Instrument	Results Intervention Period.	Results Follow-Up Period.	Boosters’ Strategies
Glazener et al. (2011) [47]	Men after prostate surgery (MAPS) Questionnaire. Practice and frequency	PR EG: 92% attended at least 1 session, and 82% attended all 4 sessions. TURP EG: 86% attended at least 1 session, and 72% attended all 4.	Over 90% returned complete questionnaires. PFMT at 12 mo.: PR: EG > CG RP EG 67%/PR CG 50%. RR 1.30, 95% CI 1.09 to 1.53. TURP: EG > CG EG: 65%/TURP CG: 20% RR 3.20, 95% CI 2.37 to 4.32.	In both EG: Booklet with instructions and reminder for PFMT and bladder training. CG no
Glazener et al. (2001) [48]	Modified version of the questionnaire [54] Frequency and amount of exercise.	No data collected in this period	Response rate questionnaire EG 75%/CG 65% PFMT performance EG > CG 79%/48% *p* < 0.001 contractions per day and Mean nº /day GE > CG 20 (29) v 5 (15), 11 to 19, *p* < 0.001	Visits at 7 and 9 months after delivery in EG. CG no
Jaffar et al. (2022) [49]	(EARS) Exercise adherence rate scale: 6 items PFMT practice (0–24) High score-high ADH. Forget/as often/do not move around/perform some, but not all, of my exercises/fit into a regular routine/perform less than recommended. KAP Q	EG: Minimal significant improvement in adherence to PFMT after 2 months of training (β = 0.033, *p* = 0.019).	No follow-up	Specific mobile APP (KEPT app) in the EG. With a progress chart and calendar.
Sacomori et al. (2020) [50]	Questionnaire[55] Time spent exercising and barriers to treatment.	No data collected in this period.	EG: 57 (85.1%) general ADH. % home-based PFMT: 32.3% 1–2 times/wk 49.3% 3–7 times/week Duration of exercise: 21 (31.3%) 3 mo. postpartum; rest 2 mo.	EG: Information leaflet for unsupervised home-based sessions.
Alagirisamy et al. (2022) [51]	Knowledge Attitude Practice (KAP) questionnaire (section practice 5 questions).	PFMT practise EG > CG (*p* < 0.001) Change from baseline in T1–T3 Significant increase in the practice score by 1.18 points (*p* = 0.038) from T1 to T3.	EG/CG marginal increase in the practice score (0, 65 points, *p* = 0.051) from the end of the 3rd trimester to the beginning of the postnatal period.	EG: Reminder messages and booster sessions. CG no
Sahin et al. (2022) [52]	100 mm visual analog scale. 0 “never completed exercises”–10 “I performed all activities”.	83%G2/91%G3 EES(G1) reported a high level of assistance.	No follow-up	Bi-weekly assistance. EES (for the EES and PFMT + EES group) was performed under the supervision of a physiotherapist 3 days a week in the clinic.
Kaya et al. (2015) [53]	100 mm visual analog scale. 10 mm increments 0% no compliance to 100% full compliance.	High in EG Bladder Training (BT) +PFMT 85% (IQR = 75, 0–100%).	No follow-up	Brief instruction sheet on BT and/or PFMT. EG (PFMT + BT) diary of exercise.

GE: experimental group; CG: control group; PFMT: Pelvic floor muscle training; ADH: treatment adherence; RP: Radical Prostatectomy; TUPR: transurethral resection of the prostate; EARS: Exercise Adherence Rating Scale; KAP Q: Knowledge Attitude Practice (KAP) questionnaire; VAS: visual analog scale; BT: Bladder Training; G1: Group 1; G2: Group 2; G3: Group 3; EES: External Electro Stimulation.

**Table 6:** Results Secondary outcomes: Symptomatic Severity–Quality of life and direct or indirect relationship with sexual health outcomes.

Study	Symptomatic Severity Pre- and Post-Data in the Intervention/Follow-Up Period	Quality of Life Pre- and Post-Data in The Intervention/Follow-Up Period	Results in Terms of Sexual Health
Glazener et al. (2011) [47]	*p* > 0.05 between groups in both interventions. Decreased improvement.	*p* > 0.05 between groups	There was no evidence from MAPS that PFMT was effective for treating sexual dysfunction.
	*p* > 0.05 between groups in both interventions. Decreased improvement.	*p* > 0.05 Improved QoL (because of the recovery from surgery)	
Glazener et al. (2001) [48]	No data collected in this period	No data collected in this period	Possible improvement of sexual health indirectly through improvement of IU-FI severity.
	Significant difference in favor of EG UI severity (X 2 = 9.49, *p* = 0.002). Use of sanitary towels (X 2 = 4.49, *p* = 0.034). Mean pad changes (t = −2.65, *p* = 0.008). Severity of FI (X 2 = 3.17, *p* = 0.075).	*p* > 0.05 in general well-being or depression. EG showed lower levels of anxiety (t = −2.08, *p* = 0.038).	
Jaffar et al. (2022) [49]	UI severity: EG improves severity in the first month (β = −4.748, *p* = 0.049), but this improvement does not persist in the 2nd mo.	QoL: no improvement is shown in participants in the 1st and 2nd mo.	Possibly no improvement in sexual health.
	No follow-up	No follow-up	
Sacomori et al. (2020) [50]	*p* > 0.05 All urinary functions declined in 3rd trimester compared with before pregnancy.	*p* > 0.05 Improvement	Possible improvement of sexual health indirectly through improvement of quality of life in both groups
	*p* > 0.05 All urinary functions improved in 3rd mo. postpartum	*p* > 0.05 (ICIQ-SF) Improvement	
Alagirisamy et al. (2022) [51]	EG significant reduction in UI severity compared with CG, which increased (Late third trimester). *p* < 0.001	Measured by self-reported ICIQ-UI-SF with severity	Possible improvement of sexual health in the EG indirectly through improvement of IU severity. Improvement was not observed in the self-reported UI
	EG > CG with an estimated mean difference of change between 0.44 and 2.54 (*p* < 0.001).	Measured by self-reported ICIQ-UI-SF with severity	
Sahin et al. (2022) [52]	Significant changes in all groups (*p* < 0.05). *p* > 0.05 between groups. All significant increases in Pelvic floor muscle strength and endurance (*p* < 0.001) but did not differ significantly among groups (*p* > 0.05)	Significant changes in all groups (*p* < 0.05) but did not significantly differ (*p* > 0.05), except for the incontinence impact subdomain (*p* < 0.05) G3 EES + PFMT > G1 EES/G3 PFMT	Possible improvement of sexual health directly through improvement of the quality of life in both groups.
	No follow-up	No follow-up	
Kaya et al. (2015) [53]	Global rating of improvement: EG > CG (100% vs. 82.7%, *p* = 0.001). Significant differences in EG for SUI (*p* = 0.001) and MUI (*p* = 0.038) but not for UUI (*p* = 0.352).	*p* > 0.05 between changes in symptom distress or QOL impact scores in MUI UUI QoL EG > CG (*p* = 0.045) SUI EG > CG Improve symptom distress (*p* = 0.001) or QOL impact scores (*p* = 0.005 and *p* = 0.040) (UDI-6 score/IIQ-7 score)	Possible improvement of sexual health in the EG indirectly through improvement of IU severity and QoL
	No follow-up	No follow-up	

UI: Urinary incontinence; SUI: Stress Urinary Incontinence; UUI: Urge Urinary Incontinence; MUI: Mixed Urinary Incontinence; FI: Fecal incontinence; EG: experimental group; CG: control group; PFMT: Pelvic floor muscle training; QoL: Quality of life; ICIQ-SF: International Consultation on Incontinence Questionnaire; ICIQ-UI-SF: ICIQ-Urinary Incontinence Short Form; EES: External Electro Stimulation; *p* > 0.05: no statistically difference.

**Table 7:** Relationship between the main and secondary outcomes.

Study	Scenario	Treatment Adherence and Compliance	Symptomatic Severity–Quality of Life
Glazener et al. (2011) [47]	1	Low adherence	No significant effect of intervention or improvement is observed in comparison with the inactive control group.
Glazener et al. (2001) [48]	3	Low adherence	Intervention favors the experimental group over the control group
Jaffar et al. (2022) [49]	NA	Showed minimal or significant improvement in adherence but did not show whether it was greater or lower than 80%.	No significant effect of intervention or improvement is observed in comparison with the inactive control group.
Sacomori et al. (2020) [50]	2	High adherence	No significant effect of intervention or improvement is observed in comparison with the inactive control group.
Alagirisamy et al. (2022) [51]	NA	Showed minimal or significant improvement in adherence but did not show whether it was greater or lower than 80%.	Intervention favors the experimental group over the control group
Sahin et al. (2022) [52]	2	High adherence	No significant effect of intervention or improvement is observed in comparison with the inactive control group.
Kaya et al. (2015) [53]	4	High adherence	Intervention favors the experimental group over the control group

Low adherence less than 80%; High adherence more than 80%; NA: not applicable.

## Data Availability

The data supporting the findings of this study can be made available upon request.

## Supplementary Materials

The following supporting information can be downloaded at: https://www.mdpi.com/article/10.3390/life15040613/s1, Figure S1: Symptomatic Severity (UI) Funnel plot for selection bias.; Figure S2: Quality of life Funnel plot for selection bias.; Table S1: Summary of the evidence of the results according to their certainty and their importance using the Grading of Recommendations Assessment, Development, and Evaluations (GRADE tool).

## Appendix A

**Table A1:** Search strategy.

**Intervention**	AND	**Participants**	AND	**Outcome**	AND	**Design**
(“Physical Therapy Modalities” OR “exercise therapy” OR “exercise” OR “Exercise Movement Techniques”)		(“Urinary Incontinence” OR “Pelvic Floor Disorders”)		(“Patient Compliance” OR “Treatment Adherence and Compliance”)		“effectiveness”

Limits in study design: randomized clinical trials or pilot study. Language limits: Spanish and English. Limits on the time of publication: no limit established. Studies published up to 15 October 2024.

**Table A2:** Specific search strategy for each database.

Database	Search Strategy
CINAHL	((((“Patient Compliance” OR “Treatment Adherence and Compliance”) AND (“Physical Therapy Modalities” OR “exercise therapy” OR “exercise” OR “Exercise Movement Techniques”) AND (“Urinary Incontinence” OR “Pelvic Floor Disorders”) AND (“effectiveness”)))) Limit “academic publications” in source type.
EMBASE	(’pelvic floor disorder’/exp OR ’urine incontinence’/exp) AND (’physiotherapy’/exp OR ’exercise’/exp) AND patient compliance/exp AND randomized controlled trial/exp
The Cochrane Plus Library	((((“Patient Compliance” OR “Treatment Adherence and Compliance”) AND (“Physical Therapy Modalities” OR “exercise therapy” OR “exercise” OR “Exercise Movement Techniques”) AND (“Urinary Incontinence” OR “Pelvic Floor Disorders”) AND (“effectiveness”)))) Limit “Randomized controlled Trial”.
Medes	(((((((((““Cooperación del paciente” “[todos]) OR ““Cumplimiento y adherencia al tratamiento” “[todos]) AND “ “Modalidades de Fisioterapia”“[todos]) OR “ “terapia por ejercicio” “[todos]) OR “ “ejercicio físico” “[todos]) OR “ “Técnicas de ejercicio con movimiento” “[todos]) AND “ “Incontinencia urinaria” “[todos]) OR ““Trastornos del suelo pélvico” “[todos]) AND ““efectividad”“[todos])
PEDro	Therapy: strength training/Problem: incontinence/Body part: perineum or genitor-urinary system/Sub discipline: continence and women’s health/Topic: no appropriate value in this field/Method: Clinical Trial
PubMed	“Patient Compliance” OR “Treatment Adherence and Compliance” AND “Physical Therapy Modalities” OR “exercise therapy” OR “exercise” OR “Exercise Movement Techniques” AND “Urinary Incontinence” OR “Pelvic Floor Disorders” AND “effectiveness” Limit “Randomized controlled Trial”.
Scopus	“Patient Compliance” OR “Treatment Adherence and Compliance” AND “Physical Therapy Modalities” OR “exercise therapy” OR “exercise” OR “Exercise Movement Techniques” AND “Urinary Incontinence” OR “Pelvic Floor Disorders” AND “effectiveness”
TRIP	“Urinary incontinence” OR “pelvic floor disorders” AND “patient compliance” OR “treatment adherence AND compliance” AND “physical therapy modalities” OR “exercise therapy” OR “exercise” OR “exercise movement techniques” Limit Key primary research.
WOS Filtered by WOS categories: rehabilitation, sport sciences, gerontology, communication, obstetrics gynecology, urology nephrology, pediatrics, cultural studies, medicine research experimental.	((((((((AB = (“Patient Compliance”)) OR AB = (“Treatment Adherence and Compliance”)) AND AB = (“Physical Therapy Modalities”)) OR AB = (“exercise therapy” )) OR AB = (“exercise”)) OR AB = (“Exercise Movement Techniques”)) AND AB = (“Urinary Incontinence”)) OR AB = (“Pelvic Floor Disorders”)) AND AB = (“effectiveness”) Limit “Clinical Trial”.

**Table A3:** Study selection.

Database	Identified	Duplicated	Design	Language	Neurological Origin	Selected for Screening	No Pelvic Floor Muscle Training (PFMT) /Intervention Based on PMFT in Both Groups/No Pelvic Floor Disorder	Eliminated After Reading the Full Text for Not Measuring Adherence or Compliance	Eliminated for Not Measuring Adherence to Treatment by Scales or Questionnaires	Included in the Review
CINAHL	556	3	509	11	1	32	29	3	0	0
EMBASE	33	11	13	0	1	8	3	4	1	0
The Cochrane Plus Library	60	41	5	0	0	14	9	2	3	0
Medes *	6	0	5	0	0	1	0	1	0	0
PEDro **	345	35	1	11	12	286	22	179	82	3
PubMed	128	10	18	2	2	96	29	50	14	3
Scopus	68	27	35	0	0	6	0	2	3	1
TRIP	150	16	37	0	0	97	87	9	1	0
WOS	85	35	31	0	0	19	12	5	2	0
TOTAL	1431	178	654	24	16	559	191	255	106	7

* Since 1 April 2024, the MEDES database has been suspended indefinitely as the Fundación Lilly wishes to promote the rest of the initiatives that make up the “MEDES-MEDicina en ESpañol project”, and to devote all its resources to them. ** In the PEDro database, this review selected studies that scored ≥4. This eligibility criterion was applied in all studies in all databases and is applied because although studies are considered good (with scores between 6 and 8) or excellent (between 9 and 10) by the scientific literature, in the particular case of the pelvic floor pathology approach, it is considered that due to the difficulty of blinding subjects and therapists, scores of 8 can be considered high quality and between 4 and 6 moderate quality [38].

**Table A4:** Timing and instruments for collecting results and Meta-Analyses.

**Treatment Compliance and Adherence**					
**Study**	**1 mo.–2 mo.**	**3 mo.**	**6 mo.**	**9 mo.**	**12 mo.**
Glazener et al. (2011) [47]		MAPS Q.	MAPS Q. Practice y—no		MAPS Q.
Glazener et al. (2001) [48]					Modified version Questionnaire [54].
Jaffar et al. (2022) [49]	EARS 21 points (KAP) Q Section 5				
Sacomori et al. (2020) [50]		Q Validated Alewijnse D. et al. 2003) [55]			
Alagirisamy et al. (2022) [51]	(KAP) Q Section 5	KAP Q (4 mo.)			
Sahin et al. (2022) [52]	100 mm VAS complete exercises (no in G3)				
Kaya et al. (2015) [53]	100 mm VAS scale. Compliance. (1.5 mo.)				
**Quality of Life**					
**Study**	**1 mo.–2 mo.**	**3 mo.**	**6 mo.**	**9 mo.**	**12 mo.**
Glazener et al. (2011) [47]		Q3 ICIQ	EQ-5D and SF-12 Q3 ICIQ		EQ-5D and SF-12 Q3 ICIQ
Glazener et al. (2001) [48]					HADS
Jaffar et al. (2022) [49]	ICIQ-LUTSqol 1 mo. and 2 mo.				
Sacomori et al. (2020) [50]		ICIQ-UI SF Q3 ICIQ			
Alagirisamy et al. (2022) [51]	ICIQ-UI SF No specific data from Q3	ICIQ-UI SF 4 mo. No specific data from Q3			
Sahin et al. (2022) [52]	KHQ baseline 2 mo.				
Kaya et al. (2015) [53]	UDI-6 + IIQ-7 baseline 6 weeks				
**Severity**					
**Study**	**1 mo.–2 mo.**	**3 mo.**	**6 mo.**	**9 mo.**	**12 mo.**
Glazener et al. (2011) [47]		ICIQ-UI SF Q1-3 p.57 pad use	ICIQ-UI SF Q1-3 pad use	ICIQ-UI SF Q1-3 pad use	ICIQ-UI SF Q1-3 pad use
Glazener et al. (2001) [48]					Question about frequency
Jaffar et al. (2022) [49]	ICIQ-UI SF 1 mo. and 2 mo.				
Sacomori et al. (2020) [50]		ICIQ-UI SF			
Alagirisamy et al. (2022) [51]	ICIQ-UI SF	ICIQ-UI SF 4 mo.			
Sahin et al. (2022) [52]	Baseline 2 mo. 24 h pad test/ ISI/UDI-6				
Kaya et al. (2015) [53]	ISI baseline and 6 weeks				
