# Supplementary material for: Compliance and Adherence to Pelvic Floor Exercise Therapy in People with Pelvic Floor Disorders: A Systematic Review and Meta-Analysis
Source: Life (Basel). 2025 Apr 6;15(4):613. doi: 10.3390/life15040613 (PMC12028920; doi:10.3390/life15040613)
Supplement: Supplementary file 1 [file life-15-00613-s001.zip › life-3560562-supplementary.pdf]

Supplementary material

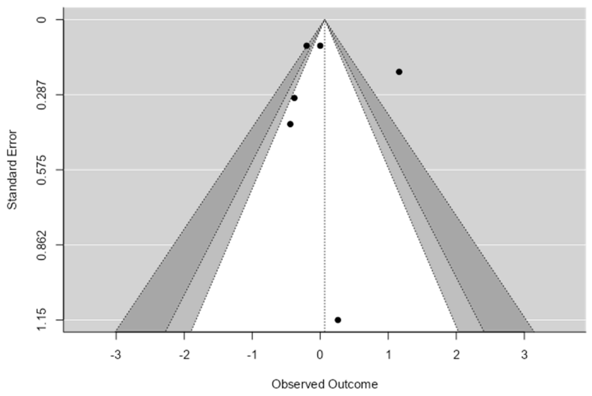

Figure S1. Symptomatic severity (urinary incontinence) Funnel Plot for selection bias.

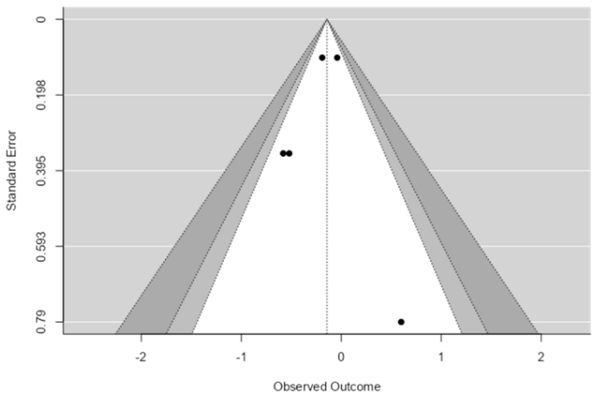

Figure S2. Quality of life Funnel Plot for selection bias.

Table S1 Summary of the evidence of the results according to their certainty and their importance using the Grading of Recommendations Assessment, Development, and Evaluations (GRADE tool).

Certainty assessment							№ of patients		Effect			
№ of studies	Study design	Risk of bias	Inconsistency	Indirectness	Imprecision	Other considerations	[Intervention] Pelvic floor muscle exercise	[Comparison] another therapeutic approach	Relative (95% CI)	Absolute (95% CI)	Certainty	Importance
**Severity (Until three months)**												
4	randomised trials	not serious	serious	not serious	not serious	none	600	597	-	SMD **0.07 SD higher** (0.48 lower to 0.61 higher)	⨁⨁⨁◯ Moderate	NO IMPORTANT
**Quality of life (Until three months)**												
3	randomised trials	not serious	not serious	not serious	not serious	none	515	512	-	SMD **0.14 SD lower** (0.28 lower to 0.01 lower)	⨁⨁⨁⨁ High	NOT IMPORTANT
**Treatment adherence and compliance**												
7	randomised trials	not serious	very serious	not serious	not serious	none	1063	1061	-	see comment	⨁⨁◯◯ Low	NOT IMPORTANT
